# Assessment of the sentiments expressed by traumatic brain injury patients and caregivers: A qualitative study based on in-depth interviews

**DOI:** 10.1016/j.heliyon.2024.e39688

**Published:** 2024-10-22

**Authors:** Raquel Mena-Marcos, Eleuterio A. Sánchez-Romero, Blanca Navarro-Main, Alfonso Lagares-Gómez-Abascal, Laura Jiménez-Ortega, Juan Nicolás Cuenca-Zaldívar

**Affiliations:** a Department of Paediatric Surgery, Hospital Universitario Vall d'Hebron, Barcelona, Spain; b Department of Rehabilitation, Faculty of Sport Sciences, European University of Madrid, Villaviciosa de Odón, 28670, Madrid, Spain; c Interdisciplinary Research Group on Musculoskeletal Disorders, Faculty of Sport Sciences, Universidad Europea de Madrid, 28670, Villaviciosa de Odón, Spain; d Physiotherapy and Orofacial Pain Working Group, Sociedad Española de Disfunción Craneomandibular y Dolor Orofacial (SEDCYDO), 28009, Madrid, Spain; e Research Group in Nursing and Health Care, Puerta de Hierro Health Research Institute - Segovia de Arana (IDIPHISA), Madrid, Spain; f Department of Psychiatry, Hospital Universitario 12 de Octubre, Madrid, Spain; g Instituto de Investigación Sanitaria Imas12, Hospital Universitario 12 de Octubre, Madrid, Spain; h Department of Neurosurgery, Hospital Universitario 12 de Octubre, Madrid, Spain; i Universidad Complutense de Madrid, Facultad de Medicina, Departamento de Cirugía, Madrid, Spain; j Department of Psychobiology, Complutense University of Madrid, 28040, Madrid, Spain; k Center of Human Evolution and Behavior, UCM-ISCIII, 28029, Madrid, Spain; l Psychology and Orofacial Pain Working Group, Sociedad Española de Disfunción Craneomandibular y Dolor Orofacial (SEDCYDO), 28009, Madrid, Spain; m Universidad de Alcalá, Facultad de Medicina y Ciencias de La Salud, Departamento de Enfermería y Fisioterapia, Grupo de Investigación en Fisioterapia y Dolor, 28801, Alcalá de Henares, Spain; n Primary Health Center “El Abajón”, Las Rozas de Madrid, Madrid, Spain

**Keywords:** Traumatic brain injury, Multidisciplinary management, Sentiments, Perspectives, Structural topic model

## Abstract

**Introduction:**

Traumatic Brain Injury (TBI) is a significant public health concern that causes death, disability, and economic burden. Its repercussions affect physical, cognitive, emotional, and behavioral aspects of long-term care needs. Despite improvements in communication among multidisciplinary teams, the management of TBI remains fragmented.

**Objective:**

This study aimed to assess patients and caregivers’ experiences through sentiment analysis.

**Materials and methods:**

A qualitative cross-sectional study utilized structured topic modeling (STM) to analyze in-depth interview data. The study involved 29 patients with TBI and 27 caregivers in Madrid (Spain), using a survey design. The interviews were conducted, transcribed, and coded independently over 5 months. Sentiments such as anticipation, fear, and emotional concerns were analyzed using three dictionaries. The STM analysis identified four key concepts: desire for independence, potential improvement, need for injury information, and psychological consequences. STM diagnostic graphs were used to determine the number of topics relevant to the evaluation of patient and caregiver concerns. Furthermore, an analysis was conducted across four topics.

**Results:**

The average age of the patients was 44.2 ± 14.9 years (69 % males). Regarding TBI severity, 59 % of patients had severe TBI, whereas the remaining 41 % had experienced moderate TBI. Among the caregivers, the majority were parents (30 %), partners (24 %), or siblings (24 % each). Among the 51 participants, sentiments were analyzed using three dictionaries. While there were no significant age differences (Z = 0.24, p = 0.815), the STM model was adjusted for significant sex differences (p = 0.017) between patients and relatives. Anticipation and fear prevailed in both groups, highlighting the shared emotional patterns.

**Discussion:**

The analysis of diagnostic graphs indicated the optimal number of topics for evaluation, emphasizing key concerns across different phases of TBI. Patients' main worries shifted from physical symptoms to limitations in daily life and independence. Caregivers highlighted the importance of staff interactions, misinformation challenges, and the need for psychological care.

**Conclusion:**

Key patient concerns, including dependency on daily activities, limitations in autonomy, and caregiver burden, emphasize crucial areas for enhancement in multidisciplinary treatment. Moreover, the lack of long-term psychological support is a significant barrier to optimal patient and caregiver well-being.

## 1 Introduction

Traumatic brain injury (TBI) is a major public health problem and an important cause of death, disability, and economic burden [1,2]. It has important physical, cognitive, emotional, and behavioral repercussions that alter individual functioning in daily life and work [3]. Moreover, the care required for these patients is not limited to acute care. Still, they may need help for the rest of their lives: many patients describe disabling symptoms months after the injury, and after normalization of neurophysiological scales, post-traumatic symptoms persist until years later [4]. However, these sequelae are multiple and have great inter-individual variation: some patients have a good recovery in a short time [5]. In addition to directly affecting patients' quality of life, it also indirectly affects their close social and family environment, with a notable loss of health-related quality of life (HRQoL) for caregivers [6]. It is estimated that 50 % of family members suffer some kind of psychological disorder due to the so-called “caregiver overload” [7]. This can lead to neglect of the patient's needs [6,7].

Caregivers of TBI patients often face a significant and prolonged burden, which can lead to physical, emotional, and psychological strain [8]. This burden is influenced by several factors, including the cognitive and functional status of the patient, with greater impairments leading to increased caregiver stress [9]. Cognitive rehabilitation plays a crucial role in alleviating some of this burden, as improvements in the patient's cognitive abilities can reduce the intensity of caregiving demands [10]. Furthermore, caregivers with higher education levels may experience heightened stress due to higher expectations and reluctance to seek external help [11]. Healthcare systems must address the substantial burden of long-term caregiving, especially for patients with ongoing dependencies [12]. Recognizing and addressing caregiver needs through educational programs and psychosocial interventions is key to improving the well-being of both caregivers and patients and ensuring better outcomes in the management of TBI [13].

TBI is a complex pathology that requires multidisciplinary management by a large number of professionals and care devices throughout its evolution [14]. However, this structure is notably fragmented in clinical practice and, although communication between all these teams is beginning to improve, there is still a long way to go towards unified management [15].

All these factors need to be considered when designing a clinical study on TBI. When analyzing the benefit/risk ratio of an intervention, several parameters or outcome measures are chosen to represent the aspects of patients' lives that will be affected by it [7,16]. In these approaches, researchers may prioritize outcomes that align with their interests, potentially overlooking those that are more important to clinicians or patients [17]. In the case of TBI, there is an added risk of excluding a fraction of the population by choosing inappropriate measurement parameters because of the clinical heterogeneity intrinsic to this pathology [18]. Focusing healthcare on outcomes can facilitate multidisciplinary work and the management of health resources, giving prominence to patients [19], provided that the outcomes measured and reported in the studies are relevant to both decision-makers and the patients themselves [7].

It is also among the GRADE recommendations to include up to Ref. [15] Patient Reported outcomes (PROs). This is supported by Cochrane reviews of the effects of health interventions and by the World Health Organization (WHO) recommendation guidelines [18], as the outcomes included in the research are not always the most relevant for patients [16].

In addition, the inclusion of PROs in the clinical practice of TBI will allow both the detection of problems or neglected needs and facilitate patient-centered care and communication, thus contributing to better health status and satisfaction on the part of health system users [20,21].

Particularly in the case of TBI, the outcome measures employed vary widely, depending on the area of application and the tool used [22]. Some are global indices, others focus only on functionality, and others focus on specific aspects or populations [23]. There is evidence in the literature on heterogeneity in the outcomes of different clinical trials, with some reviews explicitly recommending the development of an international consensus [24,25].

Despite the significant impact of TBI on the health of both patients and caregivers, it is too easy for clinical practice to pay insufficient attention to their suffering and the radical change it entails for them [26]. Most studies on functioning after TBI have been based on questionnaires, which can severely limit responses and focus on the presence of symptoms rather than the degree of impact on day-to-day life [27]. Health professionals design these measurements, often without significant input from patients or their families [5]. Patients and caregivers must be actively involved in the disease management process, especially given the importance of tailoring therapy to their needs [28].

For this reason, the present study was framed to evaluate the sentiments expressed by patients and caregivers from the main concerns detected using a Structural Topic Model (STM) analysis from deep interviews.

## 2 Materials and methods

### 2.1 Study design

A cross-sectional qualitative study based on in-depth interviews was carried out in collaboration with twenty-nine patients who had suffered TBI (ICD-10 CM code S06.30) and twenty-seven caregivers in the Community of Madrid between November 2019 and March 2020 in Madrid, Spain. All the patients were diagnosed by a neurologist at the Neurology Service of Hospital 12 de Octubre. The procedures were conducted following the Consolidated Criteria for Reporting Qualitative Research (COREQ) statement and checklist [29]. In addition to the Declaration of Helsinki, all subjects signed an informed consent form before participating in the study. This study was approved by the Ethical Committee on Clinical Research of the 12 de Octubre Hospital, Madrid, Spain Nº CEIm: 19/521.

### 2.2 Study population and data collection

A non-probabilistic purposive snowball sampling technique was used. Sampling was designed to answer the research question and was not based on the representativeness of the results [30]. For in-depth interview research, a variety of proposals are available for justifying and determining the sample size [31], and there is no formula for pre-calculating the sample size [32]. The sample size was determined following the proposal of Turner-Bowker et al. [33]. These authors suggest that 25–30 interviews per group are sufficient to identify 99 % of concepts, themes, and content. This approach enhances the ability to identify codes, categories, and themes.

In this study, 10 patients with moderate-to-severe TBI in the acute phase, 10 in the subacute phase, and 9 in the chronic were included. Patients were recruited between December 27, 2017, and January 30, 2020. Caregivers were interviewed for all but two patients: the wife of a chronic-phase patient (unsuccessful phone contact) and a patient in the acute phase with no primary caregiver. No one declined to participate.

Semi-structured interviews, guided by an open-ended script (Supplementary Material 1), were conducted with patients. Interviews were audio-recorded, verbatim transcribed, and lasted an average of 40 ± 10.4 min (total 2280 min). BNM and RMM conducted these interviews in a private hospital room. Researcher field notes provided additional context. [34]. Two code lists were obtained: one with the most relevant variables from the patients' perspective and the other from the caregivers' perspective.

### 2.3 Outcomes measures and procedure

The questions were designed to obtain as much information as possible by analyzing the knowledge of TBI. For this purpose, eight questions were devised among an interdisciplinary team of TBI experts at the Universidad Complutense de Madrid (Madrid, Spain), Universidad de Alcalá (Madrid, Spain), and the European University of Madrid, Spain (University that has the full accreditation of the World Confederation for Physical Therapy, WCPT). The questionnaire was crafted through collaborative brainstorming sessions, drawing from published literature and collective expertise. Subsequently, the questionnaire was refined via expert consensus to ensure its efficacy [35]. Two researchers (R. M. M. and B.N.M.) independently coded the interviews by initially reading and re-reading each transcript to comprehend the overall content. Subsequently, they conducted line-by-line coding independently. The researchers then compared and debated their findings through collaborative discussion. Recruitment stopped in each group when code saturation occurred and no more codes appeared.

During data collection, all interviews were conducted in the participants' native language, Spanish, to preserve the authenticity of their responses. Professional translators experienced in healthcare terminology were used to translate the transcripts into English for analysis. Additionally, dictionaries specifically adapted for Spanish were utilized to minimize the risk of data distortion. To further control for potential bias, the translated content was cross-validated against the original transcripts by two independent researchers (E.A.S.R. and J.N.C.Z.)

The analysis conducted with the Structural Topic Model and sentiment analysis dictionaries was reviewed by J.N.C.Z., a statistician specializing in Natural Language Processing. Based on his recommendations, additional details regarding the justification for the selected methodologies were incorporated into the statistical analysis section to ensure the robustness and accurate interpretation of the results.

### 2.4 Handling of personal data

The survey was executed with participant anonymity. The transcripts were pseudonymized. Following the necessary period for data quality control and analysis, all information was irreversibly anonymized and video recordings were eliminated. Data management adhered to Spanish data protection laws, including Organic Law March 2018 and Royal Decree 1720/2007 guaranteeing anonymity video recording and eliminations after publication.

### 2.5 Statistical analysis

For statistical analysis, R Ver. 4.1.3 (R Foundation for Statistical Computing, Institute for Statistics and Mathematics, Welthandelsplatz 1, 1020 Vienna, Austria).

Statistical significance was set at p < 0.05. The Kolmogorov-Smirnov test with Lilliefors correction assessed variable distributions. Descriptive statistics included means ± standard deviations and absolute/relative values (%). Fisher's exact test compared genders between patients and relatives, while the Mann-Whitney *U* test compared their ages.

The interviews were then lemmatized for analysis. Lemmatization is a linguistic process that consists of finding the corresponding lemma given an inflected form (that is, plural, feminine, conjugated, etc.). The lemma is accepted by convention as representing all the inflected forms of the same word; that is, the lemma of a word is normally the entry word in a traditional dictionary: singular for nouns, masculine singular for adjectives, and infinitive for verbs.

A Structural Topic Model (STM) analyzed topic occurrence in the interviews, accounting for gender differences between groups (covariate) The selection of the optimal number of topics was carried out in two phases: first, the range of topics with the best values of exclusivity, semantic coherence, likelihood, and the variational bound of convergence between iterations; and second, the ratio between semantic coherence and exclusivity between the selected models was analyzed. Exclusivity measures the overlap between topics, while semantic coherence assesses the consistency of words within topics. Higher values for both are desirable [[36], [37], [38]].

From each group, the probability of assigning words from the text to each of the topics was extracted by performing a sentiment analysis using the Bing [39], Afinn [40] National Research Council Canada (NRC) [41], and Stadthagen-Gonzalez [42] dictionaries. These three dictionaries are based on unigrams or individual words that assign scores for positive or negative sentiments, and the NRC dictionary classifies words into emotional categories of anger, anticipation, disgust, fear, joy, sadness, surprise, and trust. The lexicon Affin assigns words a score ranging between −5 and 5, with negative values indicating more negative sentiments and positive values indicating more positive sentiments. Sentiments were weighted by topic probability and compared across interviewee groups using the Kruskal-Wallis H test given the lack of normality.

There were no missing data as all participants answered the questions raised in the interviews.

## 3 Results

57 subjects participated in the study, 29 patients with an average age of 44,2 ± 14,9 years, mostly women (69 %) and 28 caregivers. More than half of the patients had severe TBI (59 %), and most caregivers interviewed were parents (30 %) or partners (24 %) or siblings (24 % in both cases) (Table 1). There were no significant differences between patients and relatives based on age (Z = 0.24, p = 0.815), but there were significant differences based on sex (p = 0.017) by which the STM model was adjusted.

**Table 1:** Clinical and demographic characteristics of interviewed.

Patients		
n		29
Age		44,2 ± 14,9
Gender, n(%)	Female	9 (31)
	Male	20 (69)
Traumatic brain injury classification, n(%)	Severe	17 (59)
	Moderate	12 (41)
Traumatic brain injury cause, n(%)	Traffic accident	9 (31)
	Bicycle accident	4 (14)
	Run over	2 (7)
	Quad bike accident	1 (4)
	Work accident	3 (10)
	Drop	7 (24)
	Autolytic attempt	3 (10)
Relationship of the family member interviewed, n(%)	Son/daughter	9 (24)
	Couple	9 (24)
	Father/mother	11 (30)
	Brother/sister	7 (19)
	Uncle	1 (3)
**Caregivers**		
n		28
First caregive age		47.16 ± 17.65
First caregiver gender, n(%)	Female	18 (64.3)
	Male	10 (35.7)
First caregiver study level, n(%)	Chair	1 (3.6)
	Primary	4 (14.3)
	Secondary	9 (32.1)
	University	8 (28.6)
	Vocational training	5 (17.9)
Second caregiver age		50.86 ± 15.85
Second caregiver gender, n(%)	Female	6 (21.4)
	Male	3 (10.7)
Second caregiver study level, n(%)	Primary	2 (7.1)
	Secondary	1 (3.6)
	University	3 (10.7)
	Vocational training	2 (7.1)

Data expressed with mean ± standard deviation or with absolute and relative values (%).

**Fig. 1:**
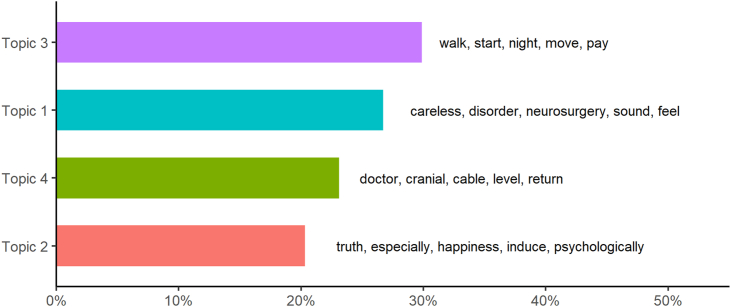
Weighted topics and most important words.

**Fig. 2:**
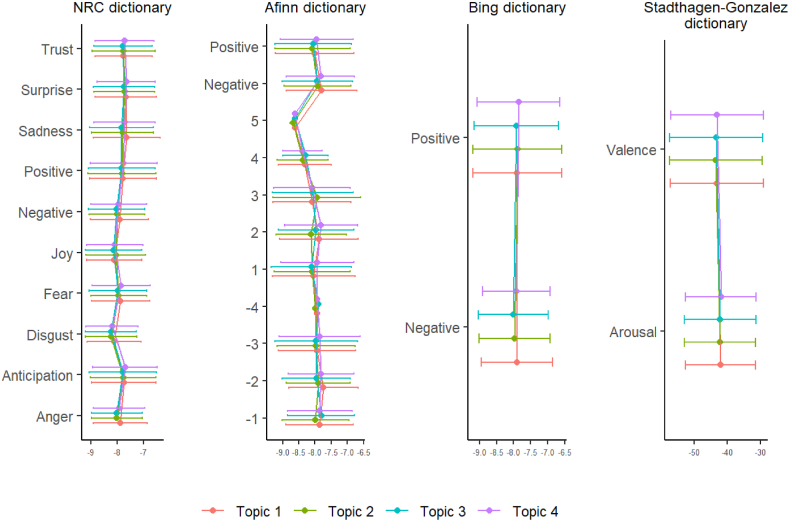
Topic sentiments weighted by word topic probability.

The STM diagnostic graphs indicated that the optimal number of topics was between two and four (Supplementary Material 2. Fig. 1), proving that the model with four topics has a better balance of exclusivity vs. semantic coherence, so the same one is chosen (Supplementary material 2. Fig. 2). Four emerging concepts were hypothesized: the desire to walk again and be independent of basic needs (topic 3), the possibility of improvement through an intervention (topic 1), the need to receive information about the injury (topic 4), and the psychological consequences of the disease (topic 2) (Fig. 1). There were no significant differences between the groups of interviewees for each of the emerging topics (Supplementary Material. Table 1).

The sentiment analysis did not show significant differences between the groups for each of the four topics (Supplementary Material 2. Table 2). When each topic was analyzed globally, a similar behavior was found in all of them, with a slight predominance, both in patients and family members, of the emotions of anticipation and fear in the NRC dictionary and negative scores in the Afinn dictionary (Table 2 and Fig. 2).

**Table 2:** Overall sentiment analysis weighted by word topic probability.

	Topic 1	Topic 2	Topic 3	Topic 4
**NRC dictionary: emotions**				
n (3954 words)				
Anger	−7.88 ± 1.02	−8.01 ± 0.97	−8.01 ± 0.97	−7.92 ± 0.99
Anticipation	−7.74 ± 1.22	−7.77 ± 1.25	−7.79 ± 1.28	−7.70 ± 1.22
Disgust	−8.11 ± 1.02	−8.23 ± 0.98	−8.23 ± 0.97	−8.19 ± 0.99
Fear	−7.87 ± 1.11	−7.94 ± 1.06	−7.97 ± 1.09	−7.85 ± 1.10
Joy	−8.11 ± 1.04	−8.05 ± 1.13	−8.13 ± 1.07	−8.09 ± 1.07
Sadness	−7.64 ± 1.27	−7.79 ± 1.17	−7.83 ± 1.20	−7.71 ± 1.16
Surprise	−7.67 ± 1.16	−7.73 ± 1.15	−7.74 ± 1.16	−7.65 ± 1.10
Trust	−7.75 ± 1.09	−7.76 ± 1.19	−7.78 ± 1.10	−7.71 ± 1.11
**NRC dictionary: valence**				
n (2562 words)				
Negative	−7.91 ± 1.10	−8.01 ± 1.05	−8.02 ± 1.06	−7.94 ± 1.05
Positive	−7.78 ± 1.27	−7.82 ± 1.29	−7.82 ± 1.26	−7.75 ± 1.26
**Afinn dictionary: scores**				
n (1326 words)				
−4	−7.94 ± 0.03	−7.99 ± 0.08	−7.90 ± 0.05	−7.93 ± 0.02
−3	−7.93 ± 1.20	−7.96 ± 1.20	−7.97 ± 1.28	−7.86 ± 1.25
−2	−7.74 ± 1.07	−7.89 ± 0.98	−7.95 ± 1.05	−7.81 ± 1.01
−1	−7.86 ± 1.04	−7.98 ± 1.03	−7.81 ± 1.02	−7.85 ± 0.98
1	−8.04 ± 1.27	−8.08 ± 1.17	−8.10 ± 1.24	−7.92 ± 1.12
2	−7.88 ± 1.21	−8.11 ± 1.08	−7.96 ± 1.16	−7.81 ± 1.12
3	−8.09 ± 1.21	−7.94 ± 1.35	−8.07 ± 1.24	−8.09 ± 1.18
4	−8.31 ± 0.82	−8.37 ± 0.78	−8.29 ± 0.70	−8.39 ± 0.61
5	−8.62 ± 0.06	−8.67 ± 0.09	−8.61 ± 0.05	−8.61 ± 0.03
**Afinn dictionary: valence**				
n (1326 words)				
Negative	−7.80 ± 1.08	−7.92 ± 1.03	−7.93 ± 1.08	−7.83 ± 1.04
Positive	−8.00 ± 1.21	−8.08 ± 1.16	−8.05 ± 1.18	−7.95 ± 1.12
**Bing dictionary: valence**				
n (2226 words)				
Negative	−7.90 ± 1.04	−7.97 ± 1.04	−8.01 ± 1.03	−7.92 ± 0.99
Positive	−7.89 ± 1.31	−7.89 ± 1.31	−7.91 ± 1.24	−7.85 ± 1.22
**Stadthagen-Gonzalez dictionary**				
n (9732 words)				
Arousal	42.03 ± 10.68	42.26 ± 10.80	42.15 ± 10.95	41.89 ± 10.66
Valence	43.24 ± 14.13	43.43 ± 14.12	43.29 ± 14.13	43.07 ± 13.97

Data expressed with mean ± standard deviation.

## 4 Discussion

Through literature analysis, expert collaboration, and patient interviews, we identified key aspects of TBI from various perspectives. Overall, 51 individuals participated, averaging 44.2 years old. Most were women (69 %), and over half experienced severe TBI (59 %). Primary caregivers were primarily parents (30 %) or partners (24 %), and siblings (24 %).

The diagnostic graphs of the STM model show that the optimal number of topics lies between two and four, with four chosen because of a better balance between exclusivity and semantic coherence. In this regard, four emerging concepts were hypothesized: desire to regain the ability to walk and independence in basic needs (Topic 3), possibility of improvement through interventions (Topic 1), need to receive information about the injury (Topic 4), and psychological consequences of the disease (Topic 2).

Regarding sentiment analysis, no significant differences were observed between groups for any of the four topics. When examining each topic globally, both patients and family members exhibited a similar pattern, with a slight predominance of anticipation and fear according to the NRC dictionary, as well as negative scores in the Afinn dictionary.

In comparison with these results, the aspects most frequently mentioned by the patients in the interviews were quite similar, with physical-physiological symptoms and cognitive and communication disorders appearing most frequently. The main difference lies in the fact that one of the main concerns of patients - and what many refer to as the biggest change they have experienced in their lives - is the transition to a situation of dependency and the limitation of performing activities of daily living with autonomy. This would correspond to the “life impact” domain, which is rarely measured as an outcome in the scientific literature [43,44]. Within the physical-physiological domain, the most relevant outcome is gait, which is directly related to autonomy in daily life.

In line with the findings of the present study, Koehmstedt et al. [45] delved into the experiences of 12 veterans and 10 caregivers following TBI. Despite not documenting the severity of TBI, researchers observed a spectrum of somatic and cognitive symptoms and psychological changes in veterans affecting work reintegration, emphasizing the crucial role of support networks, while caregivers reported stress and increased responsibilities, underscoring the need for ongoing support.

Furthermore, in the present study, clear differences were found between the reported findings according to the phase. In the acute phase, the patient's main complaints were pain and headache, followed by memory problems and post-traumatic amnesia, inability to eat and drink, and inability to get up and walk. In the subacute phase, which tended to correspond with returning home, their concerns shifted to limitations in social functions, such as walking, driving, dependency, and returning to work. Finally, in the chronic phase, concerns about cognitive impairments that prevent them from leading an independent life predominated. In this evolutionary pattern, we observed how patients' concerns appeared according to the limitations they encountered throughout the process, from hospital admission to daily life, returning to work, and interpersonal relationships.

In this regard, Linnestad et al. [46] sought to explore the experiences of people with prolonged recovery from mild TBI. Through interviews with eight participants, themes of ambiguity, hope, uncertainty about activity and rest, and the struggle to return to being themselves emerged, shedding light on the complex interplay of uncertainty and hope in the mild TBI recovery journey.

Snell et al. [47] noted the importance of having a coherent understanding of injury and recovery in 10 patients with mild TBI who were interviewed in their case-control study. The factors that facilitated coherence included social support, validation, reassurance, access to credible evidence-based information, and a pathway to wellness. In the 60-min interviews, the researchers explored participants' experiences of recovery in hospital offices or at home. Thematic analysis, which employed a general inductive approach, involved independent coding by two investigators, with a third investigator reviewing and refining the themes through collaborative discussion. This method was similar to that used in the present study, in which two researchers coded the interviews independently, first obtaining an overall understanding through repeated readings before performing line-by-line coding and then comparing and discussing their findings collaboratively.

In the context of the different phases of acute, subacute, and chronic TBI patient goes through, Togher et al. [48] developed a qualitative study on 46 and 11 women aged 16–66 years with a median age of 33 years affected by severe TBI to explore predictors of communication and psychosocial outcomes 2 years after severe TBI. They found that early cognitive performance, recovery factors, and cognitive-communicative measures at six months strongly predicted positive conversational engagement and psychosocial outcomes at two years, recommending taking these factors into account for prognostication by utilizing services during early recovery and beyond six months post-injury.

In the interviews with the caregivers, what they attached most importance throughout the process in the present study was dealing with the staff, especially in the acute phase of admission to the ICU and neurosurgery. This is fundamentally about empathy and misinformation, whether about their relative condition, prognosis, or what to do after discharge and how to seek help. Regarding the former, it is difficult to propose a strategy for improvement given the uncertainty inherent in the prognosis of TBI patients and their unpredictable evolution. Regarding the latter, the lack of coordination of care throughout the different stages of care of these patients, the need for information on the aids available, and the steps to be taken after discharge were mentioned frequently in the interviews. The authors of the present work believe that this is an important area in which to focus future improvement efforts to reduce the anxiety experienced by caregivers of these patients.

Concerning caregiver overload, mood disorders, both depressive and anxious, were frequently mentioned in all three phases. This result highlights the importance of long-term psychological care, not just during acute phases, which is currently lacking in the Spanish healthcare system. On the other hand, limited free time, especially in the subacute and chronic phases, is often due to insufficient economic support and human resources for post-discharge care. External responsibilities (not usually considered), such as work or caring for others, can further exacerbate this burden.

The results of the present study showed a parallel and some response in studies such as that of Dixon et al. [49], who explored self-efficacy in neurological rehabilitation by interviewing 24 patients with stroke, traumatic brain injury, or other neurological impairments. Eleven themes emerged, highlighting the importance of self-efficacy, determination, active participation, and collaboration with multidisciplinary teams. Patients identified complex information needs, valued goal setting, and the vicarious experiences of other patients. Recognition of personal progress and external reassurance are crucial. The study revealed two models of rehabilitation, “recovery” and “adaptation,” which influence self-efficacy. Patients drew on task mastery, vicarious experience, and social persuasion for self-efficacy, recognizing the importance of their attitudes and support received from professionals, family, and peers in the rehabilitation process.

The additional domain we have included in the present study, “Institutional-Administrative,” includes several aspects of healthcare that have influenced the experience of caregivers and patients. This was included in the study because it is a good starting point when considering possible future improvements in TBI care.

The present study identified multiple aspects of patient and caregiver health that, although they appear in the scientific literature when measuring the impact of therapeutic measures, are practically anecdotal. However, from the perspective of patients themselves, the importance of these results is diametrically opposed. This mainly refers to the patients' lack of autonomy and their limitations in daily life, as well as the caregivers' overload and impact on their mood.

Research highlights that emotional well-being significantly influences recovery in patients with TBI, with unmanaged psychological stress potentially leading to poorer outcomes, prolonged recovery times, and decreased quality of life [[45], [46], [47], [48], [49], [50]]. To enhance mental health and physical recovery, it's essential to provide psychosocial support programs and regular emotional assessments for both patients and caregivers. These study findings emphasize the importance of incorporating psychological care into integrated TBI rehabilitation models.

This study revealed strong associations between emotional stress, caregiver overload, and recovery outcomes in patients with traumatic brain injury (TBI) patients [51]. Emotions such as anticipation and fear in both patients and caregivers can negatively impact neuroplasticity and healing, whereas caregiver overload can reduce the quality of care and delay functional recovery [52,53]. Addressing these emotional factors through psychological support and improving communication can enhance patient adherence to rehabilitation and improve the overall outcomes [54]. By integrating emotional and psychological support into TBI care, autonomy, physical recovery, cognitive functionality, and quality of life can be significantly improved in both patients and caregivers [55].

Enhancing TBI management requires psychosocial support programs for patients and caregivers, as well as improved communication skills among medical staff. This will help alleviate emotional distress and provide clearer information [56]. Additionally, unifying care through multidisciplinary teams with standardized protocols and digital platforms to optimize communication has been suggested [57]. Implementing regular patient-reported outcomes (PROs) and creating specialized rehabilitation centers can enhance TBI management by enabling personalized treatment and providing comprehensive care throughout the recovery process.

### 4.1 Limitations and strengths

The study's non-probabilistic, purposive, snowball sampling approach, while focused on the research question, may limit the generalizability of the findings. Despite the larger sample size, the study may not represent the entire population. Although gender imbalances were controlled for, group differences could have influenced the observed emotional patterns. Mollayeva et al. [58] emphasized the importance of including sex as a factor in TBI research to avoid skewed data and account for societal influences. Larger sample sizes are crucial for identifying meaningful gender interactions.

The process of translating qualitative data from Spanish to English in this study may have influenced the interpretation and sentiment analysis outcomes. Language translation can lead to the loss of linguistic nuances, cultural references, and emotional tone of the original content, which are critical components in sentiment analysis and topic modeling. Such losses could potentially alter the representation of participants’ experiences and identify key themes.

Several strategies have been developed to overcome these challenges. First, professional translators with expertise in healthcare and familiarity with the context of traumatic brain injury (TBI) were employed to ensure accurate and contextually relevant translations. This approach aimed to preserve the authenticity and depth of participants’ narratives. Second, to minimize potential biases and inaccuracies, two independent researchers cross-validated the translated content against the original Spanish transcripts. This step was crucial for verifying the consistency and fidelity of the translations of the original data.

Moreover, we utilized sentiment analysis dictionaries and a Structural Topic Model (STM) tailored for bilingual text analysis. These tools were selected for their capacity to handle language-specific characteristics and provide reliable outputs despite the translation challenges. By incorporating these control measures, we aim to enhance the robustness and validity of our findings.

In line with Steigerwald et al. [59], who highlighted the benefits and limitations of translation in academic research, we acknowledge that although translation tools can bridge language gaps, they are not without flaws. Future research should continue to refine these methods, explore the use of advanced neural machine translation systems, and create multilingual glossaries specific to TBI-related topics. Such advancements would support the integration of multilingual data into sentiment analysis, ultimately enriching the representation of diverse patient and caregiver perspectives in the literature.

### 4.2 Future directions

Future studies should focus on developing comprehensive integrated care models that prioritize emotional and psychological support in TBI rehabilitation given its association with recovery outcomes. Regular assessments of emotional well-being for patients and caregivers can help monitor progress and tailor treatment strategies.

It is advisable to carry out future studies where a concrete list of outcomes that would be mandatory to measure in any study on TBI is obtained, which, by including PROs, would allow in the future to give more importance to the impact of the sequelae on the daily life of patients and caregivers and the deterioration of their quality of life due to TBI.

## 5 Conclusions

The study highlights the importance of improved coordination and the integration of patient-reported outcomes (PROs) in traumatic brain injury (TBI) management. Patients expressed concerns about dependency, autonomy, and caregiver burden, emphasizing the need for enhanced multidisciplinary treatment. The lack of long-term psychological support is a critical factor that must be addressed to improve overall well-being.

## CRediT authorship contribution statement

**Raquel Mena-Marcos:** Writing – original draft, Methodology, Investigation, Data curation, Conceptualization. **Eleuterio A. Sánchez-Romero:** Writing – review & editing, Visualization, Validation, Supervision, Methodology, Formal analysis. **Blanca Navarro-Main:** Writing – original draft, Supervision, Project administration, Investigation, Conceptualization. **Alfonso Lagares-Gómez-Abascal:** Writing – original draft, Supervision, Methodology. **Laura Jiménez-Ortega:** Writing – review & editing, Validation, Supervision, Methodology, Formal analysis. **Juan Nicolás Cuenca-Zaldívar:** Writing – review & editing, Visualization, Validation, Supervision, Software, Resources, Project administration, Methodology, Funding acquisition, Formal analysis, Data curation, Conceptualization.

## Informed consent statement

Informed consent was obtained from all the subjects involved in the study. Written informed consent was obtained from the patients for the publication of this paper.

## Institutional review board statement

The study was conducted in accordance with the Declaration of Helsinki and was approved by the Ethical Committee on Clinical Research of the 12 de Octubre Hospital, Madrid, Spain Nº CEIm: 19/521.

## Data availability statement

The data presented in this study are available upon request from the corresponding authors. The data were not publicly available because of ethical restrictions.

## Funding

This study received no external funding.

## Declaration of competing interest

The authors declare that they have no known competing financial interests or personal relationships that could have appeared to influence the work reported in this paper.

## Footnotes

Appendix A Supplementary data to this article can be found online at https://doi.org/10.1016/j.heliyon.2024.e39688.
