# Supplementary material for: Assessment of the sentiments expressed by traumatic brain injury patients and caregivers: A qualitative study based on in-depth interviews
Source: Heliyon. 2024 Oct 22;10(21):e39688. doi: 10.1016/j.heliyon.2024.e39688 (PMC11546493; doi:10.1016/j.heliyon.2024.e39688)
Supplement: Multimedia component 1 [file mmc1.docx]

**Interview script**

1. How much would you say your life has changed?
2. Throughout the process, what things have you been recovering?
3. Throughout the evolution, what has been the greatest difficulty of all?
4. And do you think she could have been helped with something else?
5. And what are the most positive things about what you have received?
6. What are the most positive things?
7. And what is the most important thing to recover in patients like you?
8. In a patient who has had a head injury, what do you think is the most important thing to support them in?
