# Supplementary material for: Assessment of the sentiments expressed by traumatic brain injury patients and caregivers: A qualitative study based on in-depth interviews
Source: Heliyon. 2024 Oct 22;10(21):e39688. doi: 10.1016/j.heliyon.2024.e39688 (PMC11546493; doi:10.1016/j.heliyon.2024.e39688)
Supplement: Multimedia component 2 [file mmc2.docx]

**Supplementary material. Figure 1:** Diagnostic model plots by topics number.


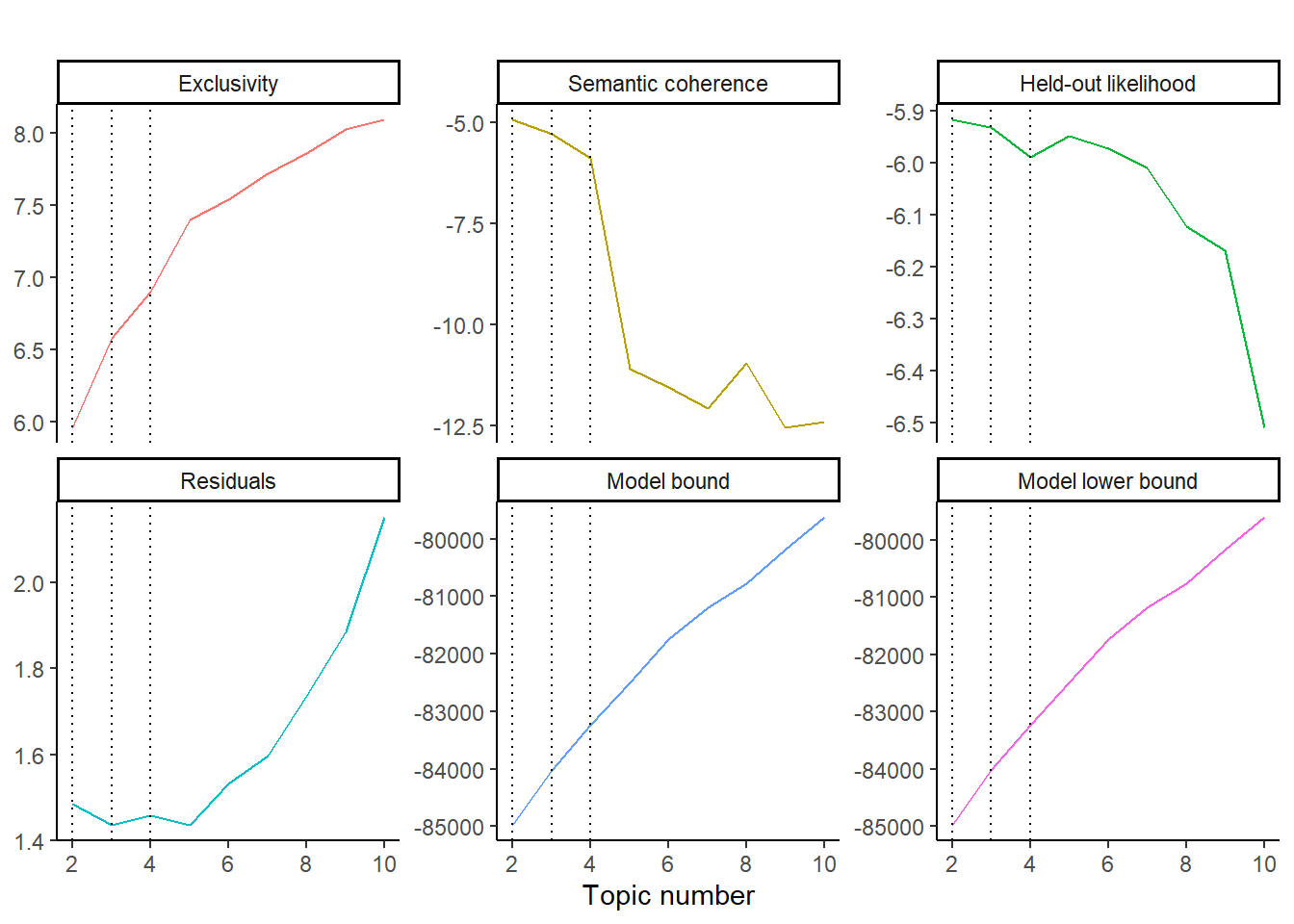


**Supplementary material. Figure 2:** Exclusivity vs. semantic coherence.


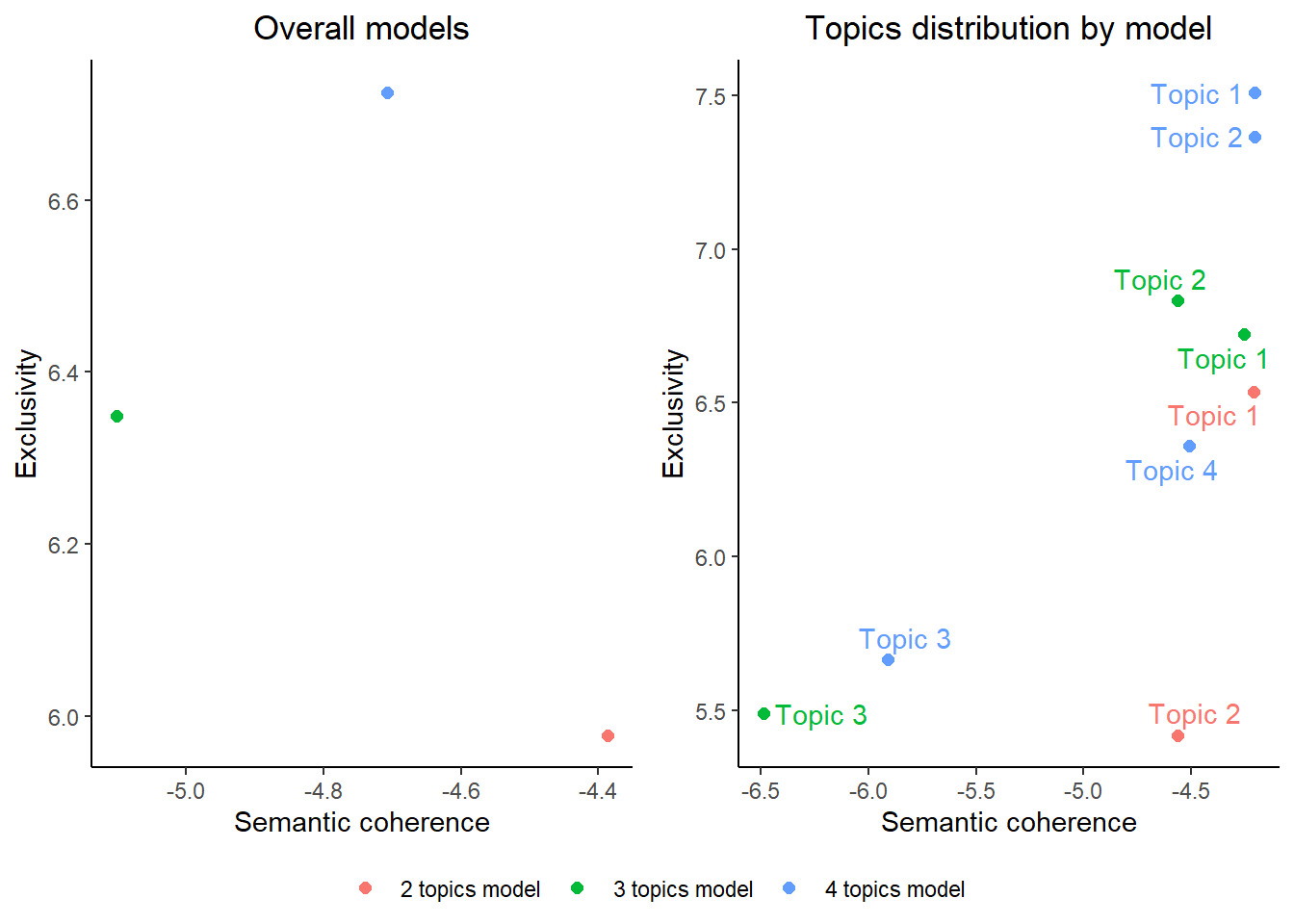


**Supplementary material. Table 1:** Topic models by group.

|  | **Coefficient (SE)** | **t statistic (^a^p value)** |
| --- | --- | --- |
| **Topic 1** | | |
| Intercept | 0.075 (SE=0.13) | 0.581, p=0.564 |
| Group (Parent-Chronic) | 0.205 (SE=0.188) | 1.092, p=0.28 |
| Group (Parent-Subacute) | -0.003 (SE=0.191) | -0.018, p=0.986 |
| Group (Patient-Acute) | 0.374 (SE=0.188) | 1.987, p=0.053 |
| Group (Patient-Chronic) | 0.303 (SE=0.187) | 1.622, p=0.112 |
| Group (Patient-Subacute) | 0.21 (SE=0.184) | 1.14, p=0.26 |
| **Topic 2** | | |
| Intercept | 0.22 (SE=0.13) | 1.686, p=0.099 |
| Group (Parent-Chronic) | -0.131 (SE=0.189) | -0.695, p=0.491 |
| Group (Parent-Subacute) | 0.11 (SE=0.191) | 0.576, p=0.567 |
| Group (Patient-Acute) | -0.086 (SE=0.186) | -0.461, p=0.647 |
| Group (Patient-Chronic) | -0.042 (SE=0.189) | -0.225, p=0.823 |
| Group (Patient-Subacute) | 0.088 (SE=0.185) | 0.475, p=0.637 |
| **Topic 3** | | |
| Intercept | 0.462 (SE=0.15) | 3.087, p=0.003 |
| Group (Parent-Chronic) | -0.101 (SE=0.213) | -0.475, p=0.637 |
| Group (Parent-Subacute) | -0.138 (SE=0.219) | -0.628, p=0.533 |
| Group (Patient-Acute) | -0.308 (SE=0.209) | -1.472, p=0.148 |
| Group (Patient-Chronic) | -0.229 (SE=0.206) | -1.115, p=0.271 |
| Group (Patient-Subacute) | -0.236 (SE=0.206) | -1.146, p=0.258 |
| **Topic 4** | | |
| Intercept | 0.238 (SE=0.136) | 1.754, p=0.086 |
| Group (Parent-Chronic) | 0.032 (SE=0.197) | 0.16, p=0.874 |
| Group (Parent-Subacute) | 0.032 (SE=0.203) | 0.157, p=0.876 |
| Group (Patient-Acute) | 0.025 (SE=0.198) | 0.125, p=0.901 |
| Group (Patient-Chronic) | -0.024 (SE=0.192) | -0.127, p=0.899 |
| Group (Patient-Subacute) | -0.059 (SE=0.193) | -0.305, p=0.762 |

Groups labeled against Parent-Acute group. SE: Standard error.

^a^significant if p<0.05 (shown in red).

**Supplementary material. Table 2:** Sentiment analysis weighted by word topic probability.

|  | **Topic 1** | | | | | | | **Topic 2** | | | | | | | **Topic 3** | | | | | | | **Topic 4** | | | | | | |
| --- | --- | --- | --- | --- | --- | --- | --- | --- | --- | --- | --- | --- | --- | --- | --- | --- | --- | --- | --- | --- | --- | --- | --- | --- | --- | --- | --- | --- |
|  | **Parent Acute** | **Parent Chronic** | **Parent Subacute** | **Patient Acute** | **Patient Chronic** | **Patient Subacute** | **ap value** | **Parent Acute** | **Parent Chronic** | **Parent Subacute** | **Patient Acute** | **Patient Chronic** | **Patient Subacute** | **ap value** | **Parent Acute** | **Parent Chronic** | **Parent Subacute** | **Patient Acute** | **Patient Chronic** | **Patient Subacute** | **ap value** | **Parent Acute** | **Parent Chronic** | **Parent Subacute** | **Patient Acute** | **Patient Chronic** | **Patient Subacute** | **ap value** |
| **NRC dictionary: emotions** | | | | | | | | | | | | | | | | | | | | | | | | | | | | |
| n (659 words) |  |  |  |  |  |  | NA |  |  |  |  |  |  | NA |  |  |  |  |  |  | NA |  |  |  |  |  |  | NA |
| Anger | -7.86±1.07 | -7.82±1.01 | -7.89±0.96 | -7.85±1.10 | -7.94±0.97 | -7.90±1.06 | 0.99 | -8.00±0.95 | -7.97±0.90 | -7.98±0.91 | -8.03±1.16 | -8.12±0.86 | -7.96±1.01 | 0.944 | -7.89±0.97 | -8.02±0.79 | -7.97±0.95 | -8.06±1.03 | -8.08±1.06 | -8.02±1.03 | 0.886 | -7.94±0.93 | -7.75±1.05 | -8.00±0.96 | -7.91±1.03 | -7.94±1.06 | -8.01±0.90 | 0.724 |
| Anticipation | -7.81±1.26 | -7.67±1.22 | -7.65±1.22 | -7.83±1.20 | -7.72±1.22 | -7.75±1.25 | 0.914 | -7.82±1.25 | -7.65±1.30 | -7.73±1.20 | -7.90±1.34 | -7.80±1.25 | -7.73±1.18 | 0.852 | -7.81±1.29 | -7.73±1.27 | -7.68±1.30 | -7.92±1.27 | -7.79±1.31 | -7.82±1.26 | 0.89 | -7.79±1.30 | -7.60±1.19 | -7.69±1.22 | -7.78±1.21 | -7.70±1.21 | -7.65±1.21 | 0.908 |
| Disgust | -8.16±1.12 | -8.15±0.87 | -8.09±0.97 | -8.10±1.13 | -8.09±0.95 | -8.05±1.14 | 0.995 | -8.25±0.93 | -8.17±1.01 | -8.24±0.91 | -8.27±1.14 | -8.33±0.85 | -8.14±1.04 | 0.934 | -7.99±1.05 | -8.24±0.92 | -8.26±0.99 | -8.27±0.98 | -8.34±0.90 | -8.28±0.95 | 0.525 | -8.24±1.01 | -8.12±0.96 | -8.15±1.09 | -8.19±1.00 | -8.20±1.02 | -8.23±0.90 | 0.989 |
| Fear | -7.80±1.15 | -7.77±1.12 | -7.87±1.00 | -7.89±1.18 | -7.96±1.04 | -7.95±1.16 | 0.805 | -7.81±1.15 | -7.83±1.09 | -7.90±1.04 | -8.09±1.07 | -8.09±0.95 | -7.94±1.05 | 0.274 | -7.81±1.06 | -7.93±1.02 | -7.79±1.24 | -8.09±1.09 | -8.14±1.03 | -8.07±1.04 | 0.097 | -7.84±1.04 | -7.66±1.14 | -7.88±1.12 | -7.87±1.15 | -7.84±1.18 | -8.00±0.99 | 0.471 |
| Joy | -8.22±1.04 | -8.04±1.03 | -8.07±0.99 | -8.15±1.07 | -8.07±1.05 | -8.09±1.09 | 0.902 | -8.12±1.04 | -7.96±1.13 | -8.04±1.04 | -8.11±1.31 | -8.02±1.23 | -8.06±1.01 | 0.958 | -8.14±1.05 | -8.04±1.09 | -8.10±1.10 | -8.22±1.05 | -8.17±1.10 | -8.10±1.04 | 0.936 | -8.18±1.10 | -8.11±0.96 | -8.05±1.12 | -8.08±1.11 | -8.11±1.03 | -8.00±1.13 | 0.94 |
| Sadness | -7.69±1.17 | -7.72±1.14 | -7.71±1.20 | -7.52±1.43 | -7.65±1.22 | -7.54±1.44 | 0.788 | -7.75±1.17 | -7.83±1.11 | -7.83±1.13 | -7.74±1.36 | -7.85±1.11 | -7.77±1.17 | 0.978 | -7.71±1.19 | -7.88±1.23 | -7.86±1.25 | -7.80±1.22 | -7.87±1.17 | -7.87±1.15 | 0.893 | -7.71±1.12 | -7.72±1.06 | -7.83±1.19 | -7.59±1.28 | -7.70±1.17 | -7.74±1.14 | 0.793 |
| Surprise | -7.67±1.17 | -7.67±1.10 | -7.68±1.08 | -7.68±1.26 | -7.67±1.20 | -7.68±1.18 | >0.999 | -7.75±1.14 | -7.69±1.10 | -7.66±1.12 | -7.85±1.26 | -7.71±1.26 | -7.71±1.05 | 0.976 | -7.68±1.11 | -7.77±1.03 | -7.75±1.19 | -7.85±1.21 | -7.70±1.31 | -7.72±1.18 | 0.98 | -7.75±1.06 | -7.49±1.17 | -7.76±1.01 | -7.71±1.12 | -7.62±1.17 | -7.55±1.12 | 0.775 |
| Trust | -7.79±1.03 | -7.76±1.02 | -7.69±1.08 | -7.78±1.11 | -7.74±1.10 | -7.75±1.19 | 0.983 | -7.74±1.19 | -7.74±1.10 | -7.74±1.05 | -7.85±1.35 | -7.73±1.29 | -7.74±1.18 | 0.969 | -7.72±1.15 | -7.73±1.10 | -7.75±1.11 | -7.86±1.11 | -7.83±1.08 | -7.76±1.04 | 0.878 | -7.72±1.15 | -7.73±1.04 | -7.73±1.11 | -7.69±1.16 | -7.66±1.09 | -7.71±1.14 | 0.996 |
| **NRC dictionary: sentiments** | | | | | | | | | | | | | | | | | | | | | | | | | | | | |
| n (427 words) |  |  |  |  |  |  | NA |  |  |  |  |  |  | NA |  |  |  |  |  |  | NA |  |  |  |  |  |  | NA |
| Negative | -7.92±1.12 | -7.92±1.02 | -7.94±0.99 | -7.85±1.22 | -7.95±1.03 | -7.87±1.21 | 0.928 | -7.98±1.05 | -7.97±1.04 | -7.98±1.02 | -8.04±1.20 | -8.12±0.94 | -7.94±1.04 | 0.596 | -7.90±1.03 | -8.04±1.00 | -7.96±1.14 | -8.03±1.13 | -8.12±1.05 | -8.09±1.01 | 0.336 | -7.96±1.03 | -7.89±1.03 | -8.01±1.03 | -7.88±1.13 | -7.96±1.08 | -7.94±1.03 | 0.841 |
| Positive | -7.84±1.29 | -7.76±1.22 | -7.72±1.26 | -7.82±1.24 | -7.75±1.31 | -7.77±1.32 | 0.936 | -7.84±1.27 | -7.76±1.24 | -7.79±1.24 | -7.90±1.37 | -7.83±1.35 | -7.78±1.28 | 0.865 | -7.84±1.26 | -7.74±1.23 | -7.82±1.27 | -7.91±1.26 | -7.85±1.29 | -7.78±1.26 | 0.811 | -7.77±1.28 | -7.76±1.17 | -7.77±1.29 | -7.77±1.26 | -7.72±1.31 | -7.72±1.27 | 0.991 |
| **Afinn dictionary: scores** | | | | | | | | | | | | | | | | | | | | | | | | | | | | |
| n (221 words) |  |  |  |  |  |  | NA |  |  |  |  |  |  | NA |  |  |  |  |  |  | NA |  |  |  |  |  |  | NA |
| -4 | -7.94 | -7.92 | -7.88 | -7.98 | -7.93 | -7.97 | NA | -7.98 | -7.92 | -7.93 | -8.13 | -8.01 | -7.95 | NA | -7.85 | -7.85 | -7.90 | -7.98 | -7.91 | -7.89 | NA | -7.92 | -7.90 | -7.95 | -7.97 | -7.92 | -7.93 | NA |
| -3 | -7.94±1.27 | -7.91±1.19 | -7.85±1.19 | -7.94±1.19 | -7.90±1.15 | -8.07±1.31 | 0.996 | -7.64±1.26 | -7.92±1.19 | -7.97±1.19 | -8.11±1.20 | -8.10±1.15 | -8.03±1.32 | 0.842 | -7.70±1.31 | -7.89±1.34 | -7.93±1.36 | -8.06±1.28 | -8.12±1.20 | -8.11±1.28 | 0.903 | -7.89±1.27 | -7.66±1.22 | -7.72±1.52 | -7.90±1.23 | -7.99±1.12 | -8.01±1.23 | 0.938 |
| -2 | -7.77±1.01 | -7.75±1.03 | -7.83±0.91 | -7.59±1.27 | -7.80±0.98 | -7.66±1.19 | 0.743 | -7.88±0.99 | -7.92±0.95 | -7.94±0.93 | -7.86±1.14 | -7.92±0.95 | -7.81±0.93 | 0.975 | -7.85±1.01 | -8.07±0.96 | -7.92±1.25 | -7.86±1.14 | -8.05±0.98 | -7.99±0.93 | 0.718 | -7.83±0.95 | -7.84±1.01 | -8.00±0.89 | -7.67±1.14 | -7.77±1.03 | -7.75±1.00 | 0.512 |
| -1 | -7.80±1.20 | -7.75±0.99 | -7.87±1.01 | -7.94±0.98 | -7.81±1.13 | -7.96±1.01 | 0.982 | -7.99±1.08 | -7.84±1.03 | -7.95±1.06 | -8.18±1.01 | -7.97±1.01 | -7.96±1.05 | 0.935 | -7.76±1.16 | -7.73±0.99 | -7.66±0.92 | -7.99±0.97 | -7.77±1.15 | -7.93±1.02 | 0.893 | -7.89±1.03 | -7.58±0.83 | -7.98±0.96 | -7.89±1.09 | -7.78±1.07 | -7.96±0.96 | 0.769 |
| 1 | -8.16±1.15 | -8.01±1.19 | -8.04±1.21 | -8.02±1.36 | -8.04±1.53 | -7.98±1.29 | 0.998 | -8.18±1.01 | -8.03±1.00 | -8.06±1.07 | -8.11±1.42 | -8.08±1.43 | -8.02±1.09 | 0.997 | -8.17±1.24 | -8.03±1.20 | -8.11±1.25 | -8.11±1.31 | -8.15±1.21 | -8.05±1.35 | 0.998 | -8.02±1.08 | -7.90±0.93 | -7.98±1.13 | -7.75±1.20 | -7.94±1.32 | -7.96±1.10 | 0.97 |
| 2 | -7.88±1.27 | -7.82±1.37 | -7.75±1.33 | -7.97±1.04 | -7.88±1.20 | -7.96±1.09 | 0.955 | -8.14±0.98 | -8.01±1.20 | -7.97±1.04 | -8.27±1.08 | -8.21±0.97 | -8.08±1.23 | 0.762 | -7.85±1.21 | -7.95±1.26 | -7.99±1.19 | -8.09±1.10 | -7.95±1.25 | -7.93±1.00 | 0.959 | -7.70±1.09 | -7.85±1.14 | -7.85±1.14 | -7.88±1.12 | -7.85±1.15 | -7.75±1.13 | 0.972 |
| 3 | -8.09±1.23 | -8.07±1.19 | -8.04±1.21 | -8.09±1.29 | -8.10±1.21 | -8.15±1.22 | >0.999 | -7.84±1.34 | -7.89±1.33 | -7.90±1.32 | -8.09±1.38 | -8.00±1.45 | -7.92±1.39 | 0.989 | -8.04±1.23 | -8.02±1.24 | -8.02±1.31 | -8.13±1.28 | -8.17±1.21 | -8.03±1.29 | 0.997 | -8.06±1.21 | -8.03±1.22 | -8.10±1.19 | -8.08±1.28 | -8.17±1.12 | -8.10±1.19 | 0.999 |
| 4 | -7.98±1.56 | -8.35±0.69 | -8.29±0.74 | -8.39±0.74 | -8.48±0.35 | -8.38±0.74 | 0.962 | -8.30±0.94 | -8.36±0.69 | -8.10±1.26 | -8.54±0.74 | -8.59±0.40 | -8.36±0.74 | 0.949 | -8.18±0.93 | -8.30±0.69 | -8.33±0.74 | -8.40±0.74 | -8.50±0.39 | -8.06±0.92 | 0.954 | -8.27±0.87 | -8.36±0.63 | -8.39±0.68 | -8.41±0.68 | -8.53±0.34 | -8.37±0.68 | 0.994 |
| 5 | -8.63 | -8.61 | -8.50 | -8.67 | -8.62 | -8.67 | NA | -8.68 | -8.62 | -8.55 | -8.83 | -8.70 | -8.65 | NA | -8.57 | -8.58 | -8.55 | -8.70 | -8.63 | -8.61 | NA | -8.61 | -8.59 | -8.57 | -8.66 | -8.61 | -8.62 | NA |
| **Afinn dictionary: sentiments** | | | | | | | | | | | | | | | | | | | | | | | | | | | | |
| n (221 words) |  |  |  |  |  |  | NA |  |  |  |  |  |  | NA |  |  |  |  |  |  | NA |  |  |  |  |  |  | NA |
| Negative | -7.81±1.08 | -7.78±1.04 | -7.84±0.97 | -7.72±1.20 | -7.82±1.03 | -7.79±1.17 | 0.973 | -7.86±1.05 | -7.90±1.00 | -7.95±0.99 | -7.97±1.12 | -7.96±0.98 | -7.88±1.02 | 0.954 | -7.80±1.08 | -7.97±1.04 | -7.87±1.20 | -7.92±1.12 | -8.00±1.05 | -8.00±1.00 | 0.687 | -7.85±1.02 | -7.76±1.01 | -7.94±1.03 | -7.76±1.13 | -7.81±1.05 | -7.84±1.03 | 0.775 |
| Positive | -8.02±1.23 | -7.96±1.24 | -7.93±1.24 | -8.04±1.17 | -8.01±1.26 | -8.04±1.15 | 0.981 | -8.09±1.08 | -8.01±1.16 | -7.99±1.12 | -8.20±1.23 | -8.15±1.21 | -8.04±1.21 | 0.752 | -8.00±1.20 | -8.01±1.20 | -8.05±1.20 | -8.13±1.17 | -8.09±1.19 | -8.00±1.15 | 0.961 | -7.91±1.11 | -7.94±1.08 | -7.98±1.13 | -7.93±1.16 | -7.99±1.16 | -7.93±1.12 | 0.994 |
| **Bing dictionary: sentiments** | | | | | | | | | | | | | | | | | | | | | | | | | | | | |
| n (371 words) |  |  |  |  |  |  | NA |  |  |  |  |  |  | NA |  |  |  |  |  |  | NA |  |  |  |  |  |  | NA |
| Negative | -7.93±1.05 | -7.88±1.02 | -7.91±0.99 | -7.82±1.13 | -7.97±0.95 | -7.88±1.11 | 0.788 | -7.95±1.02 | -7.98±0.94 | -7.98±0.99 | -7.93±1.28 | -8.09±0.93 | -7.91±1.08 | 0.547 | -7.85±1.06 | -8.05±0.98 | -7.99±1.09 | -7.98±1.08 | -8.13±0.97 | -8.03±1.00 | 0.129 | -7.89±1.00 | -7.89±0.94 | -8.01±0.99 | -7.84±1.09 | -8.00±0.92 | -7.91±1.01 | 0.495 |
| Positive | -7.93±1.32 | -7.83±1.28 | -7.86±1.30 | -7.91±1.33 | -7.93±1.36 | -7.90±1.29 | 0.981 | -7.88±1.28 | -7.84±1.24 | -7.81±1.29 | -8.00±1.34 | -7.98±1.33 | -7.82±1.37 | 0.674 | -7.89±1.25 | -7.85±1.20 | -7.94±1.23 | -7.99±1.23 | -7.97±1.25 | -7.84±1.30 | 0.84 | -7.83±1.17 | -7.85±1.15 | -7.92±1.18 | -7.86±1.25 | -7.87±1.28 | -7.79±1.29 | 0.963 |
| **Stadthagen-Gonzalez dictionary** | | | | | | | | | | | | | | | | | | | | | | | | | | | | |
| n (1622 words) |  |  |  |  |  |  | NA |  |  |  |  |  |  | NA |  |  |  |  |  |  | NA |  |  |  |  |  |  | NA |
| Arousal | -42.21±10.68 | -41.88±10.58 | -41.86±10.64 | -42.01±10.75 | -42.06±10.67 | -42.16±10.79 | 0.981 | -42.20±10.73 | -42.00±10.70 | -42.04±10.68 | -42.83±11.05 | -42.37±10.91 | -42.09±10.77 | 0.645 | -42.00±10.82 | -41.87±10.95 | -42.08±10.87 | -42.58±11.07 | -42.35±11.00 | -42.05±10.98 | 0.804 | -41.94±10.62 | -41.64±10.48 | -42.16±10.73 | -41.93±10.71 | -41.81±10.66 | -41.88±10.78 | 0.957 |
| Valence | -43.43±14.19 | -43.10±14.05 | -43.05±14.06 | -43.23±14.19 | -43.28±14.20 | -43.35±14.15 | 0.995 | -43.43±14.18 | -43.15±13.91 | -43.24±14.07 | -44.05±14.37 | -43.51±14.22 | -43.23±14.00 | 0.819 | -43.18±14.12 | -42.93±13.90 | -43.24±14.10 | -43.73±14.29 | -43.52±14.31 | -43.14±14.10 | 0.894 | -43.11±13.97 | -42.83±13.83 | -43.34±14.01 | -43.13±14.03 | -43.05±14.17 | -42.96±13.82 | 0.988 |

Data expressed with mean±standard deviation.

^a^significant if p<0.05 (shown in red).
